# Characterization and discovery of miRNA and miRNA targets from apomictic and sexual genotypes of *Eragrostis curvula*

**DOI:** 10.1186/s12864-019-6169-0

**Published:** 2019-11-12

**Authors:** Ingrid Garbus, Juan Pablo Selva, María Cielo Pasten, Andrés Martín Bellido, José Carballo, Emidio Albertini, Viviana Echenique

**Affiliations:** 1 Centro de Recursos Naturales Renovables de la Zona Semiárida (CERZOS); CONICET, Bahía Blanca, Argentina; 2 Dipartimento di Scienze Agrarie, Alimentari e Ambientali, Università degli Studi di Perugia, Perugia, Italy

**Keywords:** *Eragrostis curvula*, Apomixis, Small RNA libraries, miRNAs, miRNA target

## Abstract

**Background:**

Weeping lovegrass (*Eragrostis curvula* [Shrad.] Nees) is a perennial grass found in semi-arid regions that is well adapted for growth in sandy soils and drought conditions. *E. curvula* constitutes a polymorphic complex that includes cytotypes with different ploidy levels (from 2x to 8x), where most polyploids are facultative apomicts, although both sexual reproduction and full apomixis have been reported in this species. Apomixis is thought to be associated with silencing of the sexual pathway, which would involve epigenetic mechanisms. However, a correlation between small RNAs and apomixis has not yet been conclusively established.

**Results:**

Aiming to contribute to the elucidation of their role in the expression of apomixis, we constructed small RNA libraries from sexual and apomictic *E. curvula* genotypes via Illumina technology, characterized the small RNA populations, and conducted differential expression analysis by comparing these small RNAs with the *E. curvula* reference transcriptome. We found that the expression of two genes is repressed in the sexual genotype, which is associated with specific microRNA expression.

**Conclusion:**

Our results support the hypothesis that in *E. curvula* the expression of apomixis leads to sexual repression.

## Background

Weeping lovegrass (*Eragrostis curvula* [Shrad.] Nees) is a perennial grass of the Poaceae family, subfamily Chloridoideae [1]. This native grass of Southern Africa is found in many semi-arid regions of the world since it is able to adapt to sandy soils and drought conditions, thus representing an excellent forage resource for marginal regions [2]. The genus Eragrostis comprises more than 250 species, with *E. curvula* and *E. tef* the best studied to date [3–6]. *Eragrostis* is characterized by a basic number of x = 10 chromosomes [7]. *E. curvula* constitutes a complex that includes cytotypes with different ploidy levels (from 2x to 8x). Diploid (2n = 2x = 20) plants are rare and reproduce sexually [8]. By contrast, most *E. curvula* polyploids are facultative apomicts, although both sexual reproduction and full apomixis have also been reported [9].

In *E. curvula*, reproduction occurs via pseudogamous diplosporous apomixis [10, 11], where a megasporocyte undergoes two rounds of mitotic division to form a nonreduced tetranucleate embryo sac with an egg, two synergids, and one polar nucleus [12, 13]. Diplospory has been found in species from the Asteraceae, Solanaceae, Rosaceae, Poaceae, and Brassicaceae families, occurring in plants belonging to 68 genera [14–16]. Embryo sac development in *E. curvula* occurs via Eragrostis-type apomixis, a process specific to this grass, which contains only four nonreduced nuclei at maturity [10]. *E. curvula* embryos are parthenogenetic, but fertilization of the polar nucleus is required for endosperm development.

Apomictic reproduction involves a complex set of mechanisms that result in asexual seed production without meiosis or fertilization; thus, the resulting embryo is genetically identical to its maternal parent [17, 18]. During evolution, apomixis emerged independently multiple times, as evidenced by the variety of apomictic mechanisms described and the many occurrences of apomictic species observed in phylogenetic analyses of angiosperms [19, 20]. Apomixis is inherited as a dominant trait [17]. Although early genetic studies proposed that a single, dominant locus controls apomixis, the developmental stages of apomixis, i.e., meiotic avoidance, parthenogenesis, and fertilization-independent endosperm development, are controlled by independent loci in some species [16, 17, 21].

Some apomixis loci have been localized to low-recombination regions and appear to be associated with heterochromatin and/or substantially repetitive sequences [17]. Although such chromosomal structures and apomixis are thought to be linked, recent evidence suggests that it is more likely that these repetitive chromosomal structures occurred as a consequence of asexual reproduction and suppressed recombination, which may have evolved to maintain the genic elements required for apomixis [17].

Assuming that apomixis is a consequence of spatial and temporal changes in expression of sexual pathway-related genes [21], differential expression analysis between stages and/or tissues of sexual and apomictic genotypes represents a useful tool for unraveling the transcriptional pathways involved in this reproductive mode. Stress-like genes are expressed in aposporous initial cells in *Brachiaria brizantha* [22] and *Hieracium praealtum* [23], suggesting these genes play a role in the induction of apospory. In *B. brizantha*, these genes encode proteins including a helicase and a MADS-box transcription factor [22, 24].

Comparative analysis between apomictic and sexual *Boechera* species revealed a global decrease in gene expression within the apomict versus sexual species during the early stages of development [25]. Comparative gene expression studies of diplosporous species should focus on identifying genes involved in the avoidance of meiosis by the megaspore mother cell. Unlike the case in aposporic species, the frequency of apomixis in *E. curvula*, as evidenced by the proportion of sexual-to-apomictic embryo sacs, increases under stress conditions, such as in vitro culture, polyploidization [4], and water deprivation [26]. Several genes are thought to be involved in apomixis in this species, including methyltransferases, kinases and transposon sequences [3, 27–29], but conclusive evidence for their roles in this process is currently lacking.

The involvement of epigenetic mechanisms in the regulation and/or expression of apomixis also has been studied in various apomictic species. Apomixis is thought to result from epigenetic deregulation of the sexual pathway, thus accounting for the facultative nature of apomixis. The increase of sexual embryo sacs in *E. curvula* under different stress conditions shows that sexual and apomictic pathways coexist in facultative apomicts and that stress deregulates the silencing of the sexual one, proving the epigenetic nature of this silencing [26]. Small RNAs (sRNAs) play roles in this epigenetic mechanism, as they are huge contributors to the phenotypic plasticity of plants and are involved in plant development, reproduction, and genome reprogramming [30].

The major classes of sRNAs in plants include microRNAs (miRNAs), hairpin-derived small-interfering RNAs (hp-siRNAs), natural antisense siRNAs (natsiRNAs), secondary siRNAs, and heterochromatic siRNAs (hetsiRNAs). miRNAs are transcribed from single-stranded hairpin-folded RNA molecules known as long primary microRNAs (pri-miRNAs) by RNA Polymerase II [30]. The enzyme Dicer-like 1 initially produces small stem-loop precursor microRNAs (pre-miRNAs) from pri-miRNA, and mature miRNA duplexes consisting of the active miRNA strand and its complementary strand (typically 20 to 22 nucleotides long) are subsequently produced [30]. One strand from the initial duplex associates with an Argonaute (AGO) protein and hybridizes with target RNAs, a process guided by Watson-Crick pairing [30]. AGO-miRNA complexes are involved in post-transcriptional gene silencing via messenger RNA (mRNA) cleavage or translational repression [31]. Most miRNA genes are species- or family-specific, suggesting that they evolve rapidly and have high turnover rates [32].

*AGO* genes in sexual plants influence the number of cells that have the capacity to initiate embryo sac development [33, 34]. For example, the phenotype of maize *ago104* mutants mimics diplospory, with diploid gametes originating from a single megaspore mother cell per ovule undergoing mitosis [34]. The repression of the somatic fate of germ cells might result from the accumulation of AGO104 protein surrounding the megaspore mother cell [34]. In *E. curvula*, *in situ* hybridization studies suggest that *Ec*AGO104 could have a similar function as maize AGO104, as evidenced by its differential regulation during male and female reproductive developments between apomictic and sexual plants [29].

Several studies have focused on the expression of sRNAs in apomict and sexual plants, but no correlation between sRNAs and apomixis has thus far been conclusively established [35, 36]. The use of various types of differential gene expression analyses of small and large RNAs between apomictic and sexual individuals of a single species represents a promising approach for investigating the regulation of apomixis. Here, we developed sRNA libraries from apomictic and sexual *E. curvula* genotypes, providing a valuable tool for studying the roles of these molecules in the expression of apomixis. We identified a set of genes that were repressed in the sexual genotypes via a compatible miRNA-mRNA interaction, supporting the hypothesis that apomixis in *E. curvula* leads to sexual repression.

## Results

### Characterization of the sRNA library profiles

**Fig. 1:**
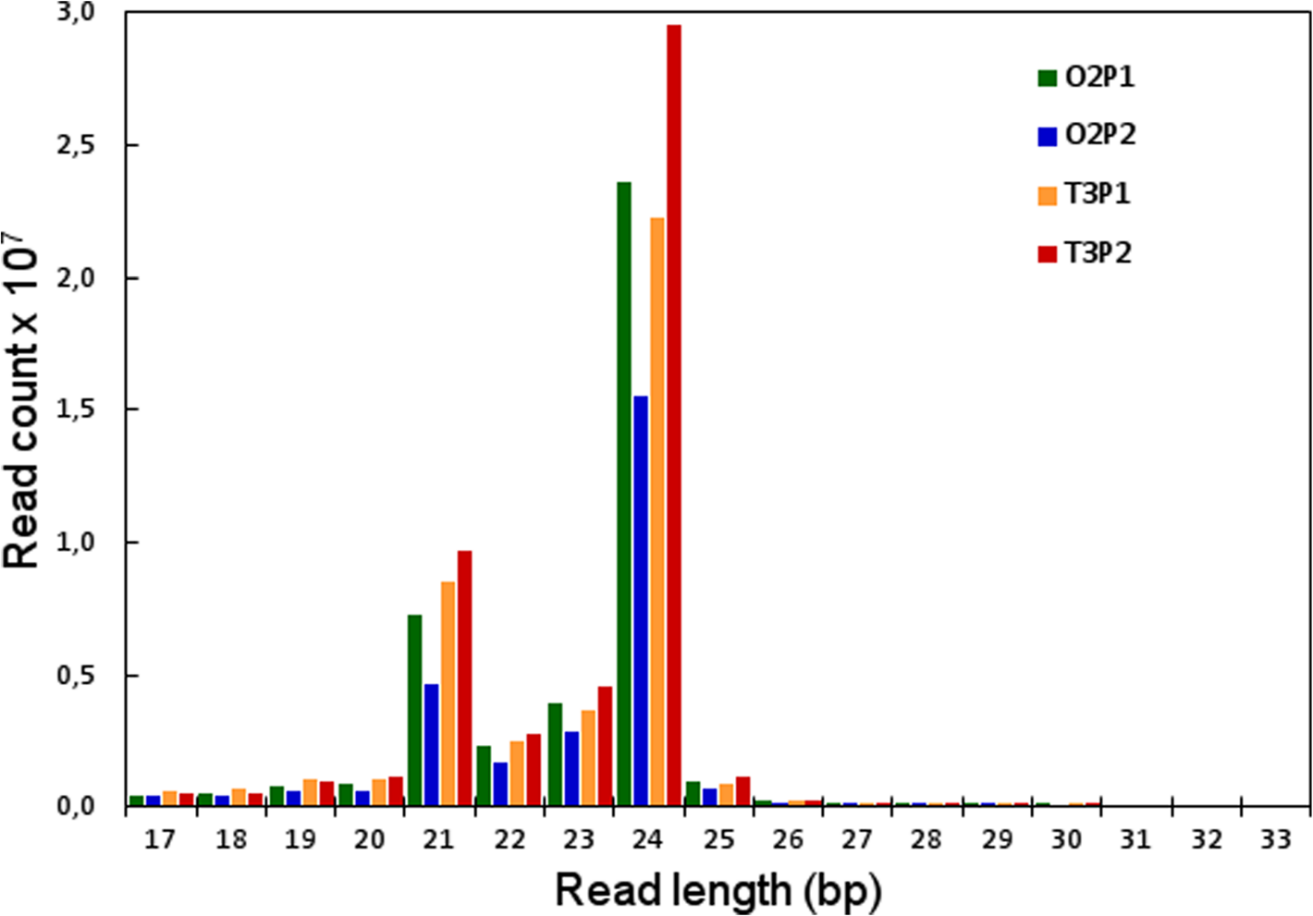
Length distribution of small RNAs in the libraries. For each reads length, the absolute read count (× 10^7^) per individual library is shown. The x-axis shows reads lengths, and the y-axis shows the frequency of occurrence of reads of each length

To elucidate the epigenetic mechanisms involved in the regulation of reproductive behavior in *E. curvula*, we developed four sRNA libraries via Illumina sequencing technology. The sRNA fraction was obtained from spikelets at all developmental stages from two *E. curvula* genotypes with contrasting reproductive modes: the apomictic cultivar ‘Tanganyika’ and the sexual cultivar ‘OTA-S’. For sequencing purposes, two biological replicates of each genotype were used and resulted in four libraries, hereafter referred to as T3P1 and T3P2 (from Tanganyika inflorescences) and O2P1 and O2P2 (from OTA inflorescences). Near ~ 40 million clean reads were generated per library, with an average length of ~ 23 bp and two peaks positioned at 21 and 24 bp, as expected (Table 1; Fig. 1). The quality scores were classified as very good (green); the percentage of adaptors, N content, GC content, and level of sequence duplication indicated that the four libraries were suitable for analysis (Additional file 1: Figure S1 and Additional file 2: Figure S2). The reads were deposited in the Sequence Reads Archive (SRA) database at NCBI as BioProject PRJNA378998 “*Eragrostis curvula* sRNA raw sequence reads” including Biosamples SAMN06564601 (biological replicates of reads from Tanganyika) and SAMN06564600 (biological replicates of reads from OTA-S). Sequence tags were labeled with the name of the library and the read count of each individual sequence. Statistics of the libraries depuration are shown in Tables 1 and 2.

**Table 1:** Description of libraries

Library	# reads	Average size (bp)	Sequence tags (minimal count = 3)	# included reads	# discarded reads	% discarded
O2P1	41,753,038	23.1	1,302,375	33,549,550	8,203,488	19.65
O2P2	28,005,457	23.0	913,356	22,244,919	5,760,538	20.57
T3P1	42,227,147	22.9	1,386,428	32,962,095	9,265,052	21.94
T3P2	51,742,747	23.0	1,658,983	40,468,525	11,274,222	21.79

For each library, the reads obtained after removing adapters and low-quality reads are shown in column 2 (# reads), followed by the average size, the sequence tag count, the number of included and discarded reads, and the percentage of reads that were discarded

**Table 2:** Statistics of the libraries depuration against sequences of chloroplast, mitochondria and RFam

Processed sequence tags	Library			
	O2P1	O2P2	T3P1	T3P2
	1,302,375	913,356	1,386,428	1,658,983
Mitochondria				
Aligned reads	2374 (0.18%)	1730 (0.19%)	4953 (0.36%)	2906 (0.18%)
Non-aligned reads	1,300,001 (99.82%)	911,626 (99.81%)	1,381,475 (99.64%)	1,656,077 (99.82%)
Chloroplast				
Aligned reads	3295 (0.25%)	2528 (0.28%)	9179 (0.66%)	3776 (0.23%)
Non-aligned reads	1,299,080 (99.75%)	910,828 (99.72%)	1,377,249 (99.34%)	1,655,207 (99.77%)
RFam				
Aligned reads	64,800 (4.98%)	48,496 (5.31%)	73,236 (5.28%)	63,895 (3.85%)
Non-aligned reads	1,237,575 (95.02%)	864,860 (94.69%)	1,313,192 (94.72%)	1,595,088 (96.15%)
Depured sequence tags	1,236,287 (94.9%)	863,918 (94.6%)	1,311,009 (94.6%)	1,593,460 (96.1%)

### Identification of conserved miRNAs

**Fig. 2:**
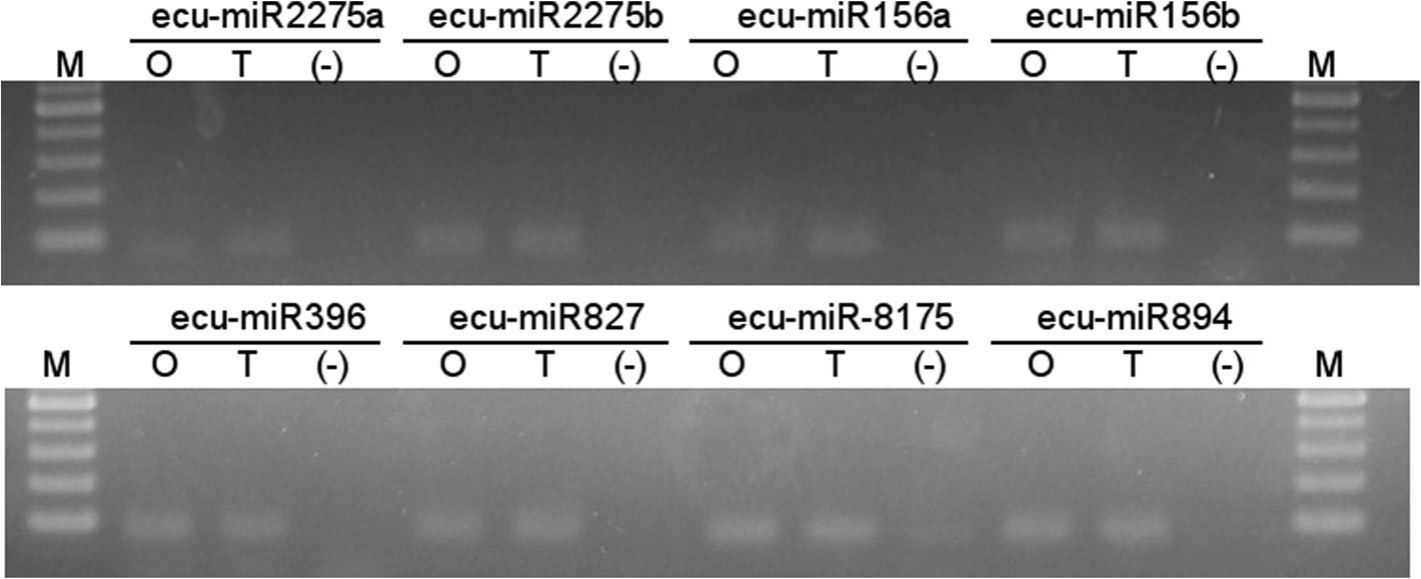
Specific amplification of conserved miRNAs from *E. curvula*. For clarity, a single run per genotype is shown. The miRNA assayed is listed above each amplification band. O, cDNA from genotype OTA-2; T, cDNA from genotype Tanganyika; (−), negative control; M, molecular weight markers. The band at the bottom is 100 bp

The sequence tags were aligned against miRBase 22.0, revealing that ~ 0.4% of each library corresponded to conserved miRNAs (Table 3a, b), for a total of 469,504 sequence tags. We analyzed the miRNAs by family, finding that ~ 75% of the sequence tags were distributed among 74 miRNA families (Additional file 4: Table S1). The greatest number of miRNAs belonged to the miR2275 and miR156 families, followed by miR396, miR827, miR169, miR5072, miR-8175, and MIR894 (Additional file 4: Table S1). We focused on the most highly conserved families and designed primers to validate their presence in biological samples (Table 4). qRT-PCR analysis of *E. curvula* miRNAs revealed miRNAs matching ata-miR2275a-3p, zma-miR2275c-3p, gma-miR156d, stu-miR156d-3p, ata-miR396b-5p, ssp-miR827, ath-miR-8175, and ppt-MIR894, which we named ecu-miR2275a, ecu-miR2275b, ecu-miR156a, ecu-miR156b, ecu-miR396, ecu-miR827, ecu-miR-8175, and ecu-miR894, respectively (Fig. 2). qRT-PCR confirmed that there were no significant differences in the expression levels of these miRNAs between genotypes, as suggested by the RT-PCR (Fig. 2).

**Table 3:** Annotation of miRNAs against miRBase v22

	O2P1	O2P2	T3P1	T3P2
a)				
Sequence tags	1,236,287	863,918	1,311,009	1,593,460
Conserved miRNAs	4953	3349	5455	6340
% of Conserved miRNAs	0.40	0.39	0.42	0.40
b)				
total count sequence tags	33,549,550	22,244,919	32,962,095	40,468,525
total count of conserved miRNAs	124,715	76,053	131,881	136,855
% of the total count of conserved miRNAs	0.37	0.34	0.40	0.34

Annotation is expressed a) in function of the unique reads annotated; and b) the total count of the cleaned reads of each library

**Table 4:** Analysis of the major miRNA families identified in the small RNA libraries from *E. curvula*

miRNA family	Database miRNA	sequence	Sequence tags found in the libraries	Count in individual libraries				In silico Differential expression	Given name	*E. curvula* conserved sequence
				O2P1	O2P2	T3P1	T3P2			
miR2275	ata-miR2275a-3p	U**UUGUUUUUCUCC**AAUAUCUCA	U**UUGUUUUUCUCC**AAUAUCUCA	–	–	1643	1266	Tanganyka	ecu_miR2275a	**UUGUUUUUCUCC**AAUAUCUC
			**-UUGUUUUUCUCC**AAUAUCUCA	331	127	400	397	–		
			U**UUGUUUUUCUCC**AAUAUCUC**-**	–	–	198	147	Tanganyka		
	osa-miR2275d	C**UUGUUUUUCUCC**AAUAUCUCA	C**UUGUUUUUCUCC**AAUAUCUCA	9465	4392	8769	9004	–		
			C**UUGUUUUUCUCC**AAUAUCUC**-**	429	213	408	301	–		
	bdi-miR2275a	U**UUGGUUUCCUCC**AAUGUCUCA	U**UUGGUUUCCUCC**AAUGUCUCA	240	–	281	257	–	–	–
	zma-miR2275a-3p	UUUGUUUUCCUCCAAUAUCUCA	UUUGUUUUCCUCCAAUAUCUCA	1826	893	1673	1477	–	–	–
			U**UUGUUUUCCUCC**AAUAUCUC**-**	494	315	367	341	–		
	zma-miR2275c-3p	U**GAGUUGGAGGAA**AAUAUCUCA	UUCAGUUUCCUCUAAUAUCUCA	2693	1012	1732	1702	–	ecu-miR2275b	UUCAGUUUCCUCUAAUAUCUC
			UUCAGUUUCCUCUAAUAUCUC**-**	698	315	378	354	–		
	ata-miR2275b-5p	A**GAGUUGGAGGAA**AACAAACA	A**GAGUUGGAGGAA**AACAAAC**C**	53	244	321	208	–	–	–
miR156	gma-miR156d	UUGACAGAAGAUAGAGAGCAC	UUGACAGAAGAUAGAGAGCAC	2643	1110	4663	5001	–	ecu-miR156a	uuGACAGAAGAuAGAGAGCAC
			UUGACAGAAGAUAGAGAGC**--**	312	182	410	451	–		
	ata-MIR156d-3p	G**CUCACUCCUCUU**UCUGUCAGC	G**CUCACUCCUCUU**UCUGUCAGCC	1084	730	953	638	–		
	stu-miR156d-3p	G**CUCUCUAUGCUU**CUGUCAUC	G**CUCUCUAUGCUU**CUGUCAUC	3526	2339	5901	8423	–	ecu-miR156b	GCUCUCUAUGCUUCUGUCAUC
	zma-miR156a-3p	G**CUCACUUCUCUC**UCUGUCAGU	G**CUCACUUCUCUC**UCUGUCAGU	335	222	547	685	–		
	bdi-miR156h-3p	G**CUCACUGCUCUU**CCUGUCAUC	GCUCACU**A**CUCUUCCUGUCA**C**C	896	530	256	323	OTA-S		
miR396	vca-miR396b-5p	U**CCACAGGCUUUC**UUGAACUA	**-**U**CCACAGGCUUUC**UUGAACUA	478	152	418	432	–	ecu-miR396a ecu-miR396b	UCCACAGGCUUUCUUGAACU CCACAGGCUUUCUUGAACUG
	sbi-miR396e	UU**CCACAGGCUUUC**UUGAACUG	UU**CCACAGGCUUUC**UUGAACUG	379	144	299	292	–		
	fve-miR396e	UU**CCACAGGCUUUC**UUGAACU	**-**U**CCACAGGCUUUC**UUGAACUU**-**	252	76	261	176	–		
	ata-miR396b-5p	U**CCACAGGCUUUC**UUGAACUG	**GCCACAGGCUUUC**UUGAACUG	235	103	209	179	–		
			**A**U**CCACAGGCUUUC**UUGAACUG	454	151	380	340	–		
			U**CCACAGGCUUUC**UU**U**AACUG	174	51	134	126	–		
			**G**U**CCACAGGCUUUC**UUGAACUG	410	137	370	345	–		
			UCCACAGG**A**UUUCUUGAACUG	202	50	108	99	–		
			U**CCACAGGCUUUC**UUG**C**ACUG	46	118	158	108	–		
	ata-miR396d-3p	GUUCAAGAAAGCCCAUGGAAA	GUUCAAGAAAGCCCAUGGAAA	114	64	142	163	–		
	osa-miR396e-5p	UCCACAGGCUUUCUUGAACUG	**--**UCCACA**A**GCUUUCUUGAAC**--**	356	257	53	57	OTA-S		
	osa-miR396d	U**CCACAGGCUUUC**UUGAACGG	UCCACA**A**GCUUUCUUGAACGG	312	93	52	57	OTA-S		
miR827	ssp-miR827	UUAGAUGACCAUCAGCAAACA	UUAGAUGACCAUCAGCAAACA	1751	1229	2565	3373	–	ecu-miR827	UUAGAUGACCAUCAGCAAACA
			UUAGAUGACCAUCAGCAAAC**-**	166	126	205	278	–		
			**-**UAGAUGACCAUCAGCAAACA	47	29	63	98	–		
	bdi-miR827-5p	UUUUGUUGGUUGUCAUCUAACC	UUUUGUUGGU**G**GUCAUCUAACC	261	229	627	563	–		
miR169	bdi-miR169l	UAGCCAAGGAUGAAUUGCCGG	CAGCCAAGGAUGAAUUGCC	243	117	27	28	OTA-S		
			CAGCCAAGGAUGAAUUGCCGG	4061	1758	397	570	OTA-S		
	vvi-MIR169y	UAGCGAAGGAUGACUUGCCUA	CGAGCUUGUUUACCGUUGAUAC	250	143	98	81	–		
			GUAUCAACGGUAAACAAGCUCGG	139	76	32	50	–		
miR5072	osa-miR5072	CGAUUCCCCAGCGGAGUCGCCA	**------**CCCCAGCGGAG**T**CGCCA	419	192	438	495	–		
			CG**-**UUCCCCAGCGGAGUCGCCA	220	188	187	276	–		
			**---**UUCCCCAGCGGAGUCGCCA	225	137	238	254	–		
			**----**UCCCCAGCGGAGUCGCCA	425	199	294	454	–		
			**--G**UUCCCCAGCGGAGUCGCCA	189	157	169	231	–		
			**---**GUCCCCAGCGGAGUCGCCA	94	63	104	127	–		
			**--**GAUCCCCAGCGGAGUCGCCA	71	64	66	78	–		
			**UCG**UUCCCCAGCGGAGUCGCCA	493	270	376	–	–		
miR-8175	ath- miR-8175	G**AUCCCCGGCAAC**GGCGCCA	CCCCGGCAACGGCGCCA	297	224	727	623	–	ecu-miR8175	UCCCCGGCAACGGCGCCA
			CG**AUCCCCGGCAAC**GGCGCCA	103	–	79	107	–		
			**UCCCCGGCAAC**GGCGCCA	293	154	488	377	–		
			UUCG**AUCCCCGGCAAC**GGCGCCA	56	27	54	42	–		
			UUCG**GUCCCCGGCAAC**GGCGCCA	27	9	35	38	–		
			UUCG**UUCCCCGGCAAC**GGCGCCA	99	42	98	99	–		
MIR894	ppt-MIR894	CGUUUCACGUCGGGUUCACC	**UU**CGUUUCACGUCGGGUUCACC**A**	148	157	127	237	–	ecu-miR894	GUUUCACGUCGGGUUCACCA
			**U**CGUUUCACGUCGGGUUCACC**A**	223	433	290	758	–		
			GUUUCACGUCGGGUUCACC**A**	238	241	242	249	–		
			UUUCACGUCGGGUUCACC**A**	353	543	1028	382	–		
			UUCACGUCGGGUUCACC**A**	1855	1718	1363	1222	–		
			UCACGUCGGGUUCACC**A**	1873	1710	1783	1468	–		

The analysis is based on the identity of the miRNAs with miRNAs from miRBase. The miRNAs sequences differentially expressed in silico are indicated by the genotype name. The last columns show the names of the E. curvula miRNAs based on the names of miRNAs from the database and the consensus sequence obtained. Oligonucleotides corresponding to these sequences were used as primers to amplify sequences from cDNAs of the conserved miRNAs

### Identification of miRNA targets

Given the lack of information about *E. curvula* miRNAs and its genome, which is restricted to the sexual diploid Victoria [5], we aimed at identifying the miRNAs based on their possible targets.

A huge amount of possible targets were retrieved in the interface psRNATarget (http://plantgrn.noble.org/psRNATarget/) [37] output. Due to our inability to validate such a large set of data, we selected several miRNAs and their targets, focusing on the possible differential representation of miRNAs and/or transcripts according to the reproductive mode of the plant and the function of the encoded protein.

**Fig. 3:**
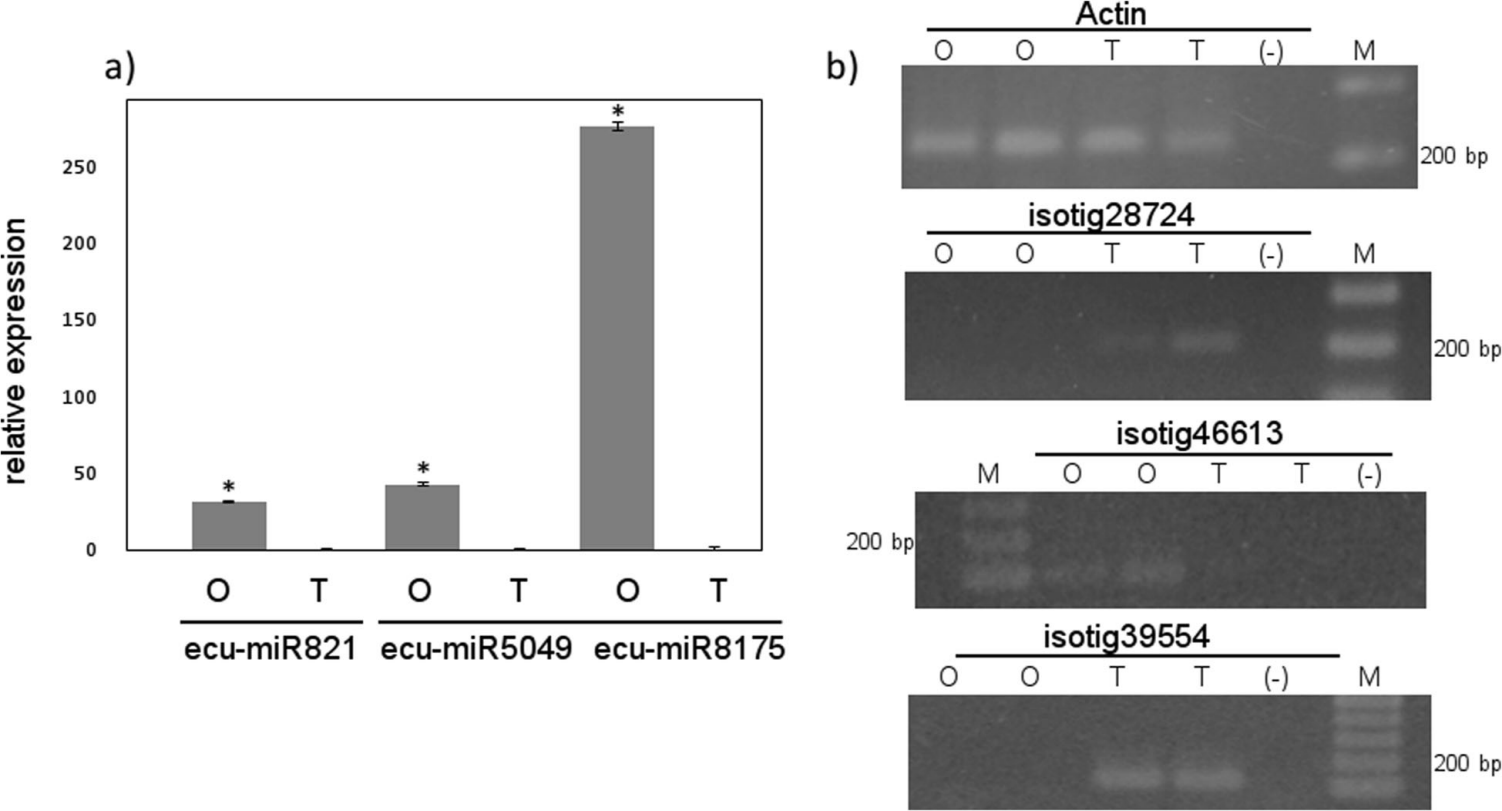
Expression of novel miRNAs and predicted targets. **a** Quantification of miRNAs via qRT-PCR; **b** amplification of the mRNAs targeted by the miRNAs by RT-PCR. O, cDNA from genotype OTA-2; T, cDNA from genotype Tanganyika; (−), negative control; M, molecular weight markers. *: *p* < 0.01

Two novel miRNA, GAACTGTTAGAGTTTGCCGCG and TATATTTTGAAACAGAGGGAG that shares partial identity with osa-miR812k and hvu-miR5049b, respectively and thus were named ecu-miR821 and ecu-miR5049, and a third one, ecu-miR8175, were found to be in silico differentially expressed in the sexual genotype OTA-S (logFC > |2|; *p* value < 0.01) and was further validated on cDNA samples by qRT-PCR (Fig. 3a). Our interest was focused on these miRNAs since the first one targets MADS-box transcription factor 6 (encoded by isotig28724) that was expressed in the apomictic genotype and the second one targets an uncharacterized transcript coded by isotig46613, only detected in cDNA obtained from OTA-2 (Fig. 3b), suggesting that the regulation of the expression of this mRNA could not be attributed exclusively to this miRNA. The third one, ecu-miR8175, targets isotig39554 (GFVM01039049.1), encoding a transposable element protein, expressed exclusively in the apomictic genotype Tanganyika (Fig. 3b).

**Fig. 4:**
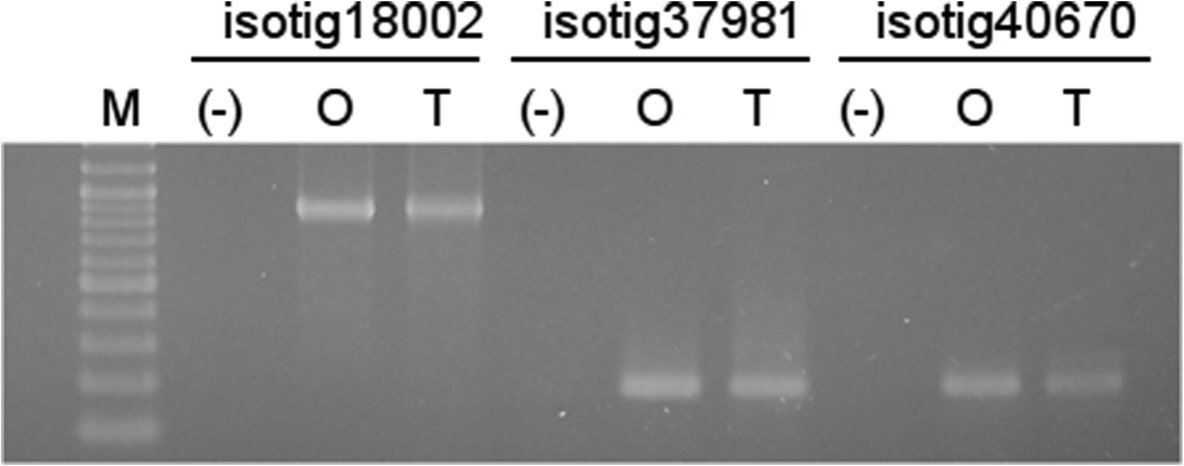
Genomic amplification of the isotig18002, isotig37981, and isotig40670 loci. O, cDNA from genotype OTA-2; T, DNA from genotype Tanganyika; (−), negative control; M, molecular weight markers. The band at the bottom is 100 bp

We searched for targets of the conserved miRNAs and analyzed a subset of mRNA targets. Among the conserved miRNAs that targeted *E. curvula* transcripts, the greatest numbers belonged to the miR156 and miR8175 families. Two members of the miR156 family, ecu-mir156a and ecu-mir156b, were identified in our libraries, which share identity with gma-miR156d and stu-miR156d-3p, respectively (Table 4). ecu-mir156a, which targets isotig18002, isotig37981, and isotig40670, encodes a squamosa promoter-binding-like (SPL) protein. These targets were successfully amplified from OTA-2 and Tanganyka genomic DNA (Fig. 4). However, no amplification product was obtained when we assayed biological replicates of cDNAs from both genotypes, which is in agreement with the notion that ecu-miR156a (Fig. 2) plays a role in regulating mRNAs predicted to be isotig18002, isotig37981, and isotig40670.

Other targets of the conserved miRNAs identified through the interface psRNATarget include the following: ecu-miR396 targets isotig19537 (encoding a growth-regulating factor family protein); ecu-miR827 targets isotig22795 (encoding an SPX domain-containing membrane protein); ecu-miR2275a targets isotig16120 (encoding a membrane-anchored ubiquitin-fold protein); ecu-miR2275b targets isotig35874 (encoding a 3-phosphoinositide-dependent protein kinase). No mRNA targets were identified for ecu-mir894.

## Discussion

Apomixis has emerged independently multiple times during angiosperm evolution, resulting in various apomictic mechanisms [19, 20]. These mechanisms might involve epigenetic silencing of the sexual pathway or, as recently reviewed by [38], of the apomictic one, if the apomictic state is considered to be the baseline, with its silencing having allowed the development of sexuality. In both cases, the epigenetic silencing could be accounted for by the facultative nature of apomixis. Since miRNAs contribute to the phenotypic plasticity of plants and are involved in development, reproduction, and genome reprogramming [30], they represent exciting candidates for studying the modulation of the expression of apomixis in *E. curvula*. The existence of natural sexual and apomictic tetraploid genotypes makes this species an invaluable model system for studying apomixis, particularly considering the Eragrostis-type of diplosporous apomixis described in *E. curvula* [13]. Elucidating the mechanisms underlying apomixis in *E. curvula* would have a strong impact on agriculture at the global level, as it would provide molecular tools to facilitate the transfer of apomixis to species of agronomic interest. The scientific interest in *E. curvula* prompted us to provide EST [39] and full transcriptome data [3] for this species to the scientific community.

The possible link between sRNAs and apomixis remains unclear. Therefore, differential expression analysis combining small and large RNAs from *E. curvula* genotypes with contrasting reproductive modes represents a valuable approach for investigating the mechanism regulating apomixis. In the present study, we produced sRNA libraries from the spikelets of apomictic and sexual genotypes of *E. curvula* via Illumina sequencing technology, which met the quality criterion needed to be used for sRNA characterization in this species. To the best of our knowledge, this is the first available set of sRNA data for this species, which has the added benefit of being obtained from genotypes with contrasting reproductive modes. This dataset, combined with previous transcriptomic data, allowed us to identify conserved miRNAs and their targets.

As expected, when we compared the sequences to those in miRBase, a small proportion (~ 0.4%) of the sequence tags showed identity with miRNAs from the database and were further classified as conserved miRNAs. A total of 75 miRNA families covered 75% of the matching sequence tags, whereas other miRNA families were not as well represented in *E. curvula*. Moreover, nearly 10% of the miRNAs from miRBase were detected in our libraries, suggesting that non-conserved miRNAs could play roles in the regulation of translation in *E. curvula*. Most of the miRNAs belonged to the miR2275 family, which trigger the biogenesis of 24-nucleotide phased sRNAs [40], as well as the miR156 family; these miRNAs are mainly expressed during the early stages of shoot development and are involved in repressing the transition from the juvenile to the adult phase of vegetative development by inhibiting the cleavage of their target SPL protein [41]. At the stages included in this analysis, ecu-miR156a was expressed in both *E. curvula* genotypes. Given that SPL genes were amplified from genomic DNA, the most plausible explanation is that ecu-miR156a is repressing the expression of the mRNA encoding SPL. Interestingly, seven additional transcripts encoding SPL isoforms were identified in the reference transcriptome, but they are not targeted by miR156, as revealed by analysis using the psRNATarget interface.

Based on its similarity to the transposable element protein retrotrans_gag (ABA97988.1), isotig39554 (GFVM01039049.1) is a putative transposable element; its expression was repressed in the sexual genotype by ecu-miR8175 overexpression, whereas it was expressed in the apomictic genotype. Ecu-miR8175 expression is modulated under various conditions [42–44], but this is the first time that a link was proposed between the differential expression of this miRNA and the reproductive mode. Previously reported targets for miR8175 include an aquaporin-like superfamily gene in barley [42], an ABA-dependent kinase gene in *Arabidopsis thaliana* [45], and several unrelated genes in *Jatropha curcas* [46], suggesting that even when a miRNA is conserved across species, its roles can vary.

## Conclusions

In the present study, we explored the role of miRNA-mRNA interactions in apomixis in the grass *E. curvula* using a combination of previously acquired transcriptomic data [3] and newly developed small RNA libraries. Supporting the hypothesis that apomixis results from the deregulation of the sexual pathway, we found that two genes, a MADS-box transcription factor gene and a transposon, were specifically repressed in the sexual genotype, most likely due to interactions with miRNAs. These results strongly suggest that miRNA is involved in the expression of apomixis in *E. curvula* via its repressive effects on gene expression. Further studies are needed to identify the link among these genes.

## Methods

### Plant materials

Two tetraploid accessions of *Eragrostis curvula* (weeping lovegrass) provided by the United States Department of Agriculture (USDA) were used in this work: the diplosporous apomictic genotype ‘Tanganyika’ (USDA: PI234217) (2n = 4× = 40) and the sexual genotype ‘OTA-S’ (USDA: PI574506) (2n = 4× = 40). Both genotypes were grown in a greenhouse under natural light conditions at 25 °C.

### RNA extraction and sequencing

Four RNA samples were prepared: O2P1 and O2P2 were biological replicates derived from spikelets with basal flowers at the beginning of anthesis from two plants of the sexual genotype OTA-S; T3P1 and T3P2 were biological replicates obtained from spikelets of two plants of the apomictic genotype Tanganyika. For both genotypes spikelets with basal flowers at the beginning of anthesis were collected for RNA extraction based on the reproductive calendar showed in Selva et al., [29] where all developmental stages are represented, from early archespores to mature female gametophytes. The development of the *E. curvula* spikelet is typically heterochronic. Basal flowers within a spikelet are more developed than flowers at the top of the spikelet. In sexual genotype, from the top to the base we found flowers containing ovules at archesporial cell, megaspore mother cell, embryo sacs with bi, tetra and octanucleate stage and mature embryo sacs with proliferating antipodal cells. Apomictic genotype shows archesporial cell, megaspore mother cell, megaspore mother cell elongated, bi and tetranucleate stage and mature embryo sac without antipodal cells.

For each sample, 30 mg of fresh tissue was ground to a fine powder in liquid nitrogen. Total RNA was extracted from the plant tissue as two fractions, small and large RNA, including RNA sequences smaller and larger than 200 bp, respectively, using a commercial RNA purification kit (Macherey-Nagel) according to the manufacturer’s instructions. The RNA was resuspended in diethylpyrocarbonate (DEPC)-treated water. The large RNA fraction was used for cDNA library construction [3], and the sRNA fraction was quantified by UV spectrometry (260 nm). The amount of sRNA ranged from 0.8 to 1.4 μg. sRNA library preparation and sequencing were conducted by GenXPro GmbH (Germany). Briefly, RNA was successively ligated to modified 3′ and 5′ adapters (TrueQuant RNA adapters, GenXPro), followed by reverse transcription and PCR amplification using specific primers. The amplified libraries were size-selected by polyacrylamide gel electrophoresis. The miRNA population, containing flanking p5 and p7 adapters, was sequenced on a HiSeq 2000 (Illumina).

### Bioinformatic analysis

After trimming the reads for adaptors and low-quality sequences as well as sequences shorter than 17 nt and longer than 33 nt, the occurrence of each unique sequence read was counted. The four individual libraries were separately collapsed by aligned using Bowtie (1.1.2) under the following parameters: --wrapper basic-0 -f -v 0 -a --best --strata -S --sam. Following the statistical criteria generally accepted for sRNA library analysis, sequence tags with counts < 3 were discarded. The remaining sequence tags were compared with the sequences of noncoding RNAs families (rRNA, tRNA, snRNA, and snoRNA) via alignment using Bowtie against the Rfam database (version 12.2) to identify degradation fragments of noncoding RNAs. Sequence tags sharing identity with mitochondrial and chloroplastic DNA sequences were also discarded.

### Identification of conserved miRNAs

To identify and annotate conserved miRNAs, the four sequence datasets obtained after the bioinformatics analysis described above were mapped onto the miRNA precursors and mature miRNAs from miRBase (release 22, http://www.mirbase.org/) using local BLAST without gaps and a maximum number of mismatches of 2, excluding hits with coverage < 90%. Unique reads that mapped to known miRNAs were classified as conserved miRNAs.

### Identification of novel miRNAs and mRNA targets

Reads were classified as novel miRNAs based on the identification of their possible targets. The sequence tags from the four libraries were subjected to BLAST analysis against the reference transcriptome, allowing no mismatches and no gaps. Sequence tags that matched each transcript were graphed and manually analyzed. When a transcript showed reads targeting across its entire length, the reads were considered to be degradation products and were discarded as possible novel miRNAs. The remaining sequence tags were used as input for further analysis through the interface psRNATarget (http://plantgrn.noble.org/psRNATarget/) [37] with the following parameters: 1) maximum expectation: 2.5 (range: 0–5.0); 2) length for complementary scoring (hsp size): 20 (range: 15–30 bp); 3) target accessibility – allowed maximum energy to un-pair the target site (UPE): 20 (range: 0–100); 4) seed region: 2–13; 5) mismatches allowed in the seed region: 0; 6) flanking length around target site for target accessibility analysis: 17 bp upstream/13 bp downstream; 7) range of central mismatch leading to translational inhibition: 9–11 nt; and 8) bulges on the target sequence in the alignment with miRNA not allowed. Previous trimming of degradation products reduced the number of possible miRNA candidates, thereby increasing the accuracy of the predicted data.

### Quantitative RT-PCR (qRT-PCR) and stem-loop qRT-PCR

For the validation assays, total RNA was isolated from Tanganyika and OTA-S spikelets collected under the same criteria used to obtain the samples for sequencing. However, an alternative, two-phased protocol was used for RNA isolation. The first phase involved a modified cetyltrimethylammonium bromide (CTAB)-based protocol [47]. The tissues (~ 35 mg) from two biological replicates per sample were homogenized in liquid nitrogen and combined with CTAB extraction buffer (100 mM Tris HCl, pH 8, 1.4 M NaCl, 20 mM EDTA pH 8, 2% [w/v] CTAB, and 0.5% β-mercaptoethanol [v/v]). After incubation at 65 °C for 10 min, chloroform was added and the sample was centrifuged at 12,000 x g (10 min). RNA was precipitated from the aqueous phase overnight on ice using 3 M LiCl, in the second phase, samples obtained in phase I were mixed with TRIzol™ Reagent (Invitrogen). Following the addition of chloroform, the homogenate was separated into three phases via centrifugation. RNA was precipitated from the upper layer with isopropanol and washed with ethanol. The samples were resuspended in 20–25 μl of DEPC water and stored at − 80 °C. The RNA was quantified using a DeNovix spectrophotometer and its integrity checked by agarose gel electrophoreses.

The validation of the conserved miRNAs was carried out as follows: cDNA was synthetized from 3 μg of total DNA-free RNA per sample using a Mir-X miRNA First-Strand Synthesis kit (Clontech Laboratories, Inc.) following the manufacturer’s recommendations. Basically, in a one-step reaction, RNA was polyadenylated and reverse transcribed using poly(A) polymerase and SMART® MMLV. The cDNA working solutions were obtained from stocks via 10-fold dilution. To measure conserved miRNA expression, with the predicted conserved miRNAs used as forward primers and the reverse primers provided in the Mir-X miRNA First-Strand Synthesis kit (Table 5). RT-PCR was conducted in a 25-μL reaction volume containing 1.5 μl cDNA template, 0.25 μM of each primer, 0.8 mM dNTPs, 1.5 mM MgCl_2_, 1X Taq buffer, and 1 U Taq polymerase (Invitrogen, Brazil) using a MyCycler Thermal Cycler (Bio-Rad, Hercules, CA, USA). The cycling conditions were as follows: 1) denaturation step, 2 min at 94 °C; 2) 38 cycles of 94 °C for 15 s, 20 s at the optimal annealing temperature for each primer pair, and 72 °C for 30 s; and 3) extension step, 72 °C for 3 min. The amplification products were separated by 2% (w/v) agarose gel electrophoresis, visualized after staining with ethidium bromide under UV light, and imaged with a Kodak Easy share Z7590 zoom digital camera. Quantitative reverse-transcription PCR (qRT-PCR) was conducted in reactions including 50 pmol of forward and reverse primers, 5 μl of 100-fold diluted cDNA, and 10 μl of Real Mix (Biodynamics, Buenos Aires, Argentina). Amplifications were carried out in a Rotor Gene 6000 (Corbett Research, Sydney, Australia). Cycling consisted of 94 °C for 2 min, followed by 45 cycles at 94 °C for 15 s, 20 s at the optimal annealing temperature for each primer pair, and 72 °C for 30 s. The specificity of the amplification products was verified by melting curve analysis performed using 1-s cycles from 72 to 95 °C, increasing the temperature by 0.5 °C per cycle. Actin housekeeping gene was amplified using *E. curvula*-based primers (F: 5′- AATGAGCTCCGTGTAGCACCAGAA-3′; R: 5′-ACATGGCTGGAACACTGAAGGTCT-3′) as described previously [27].

**Table 5:** Primers designed for PCR amplification

Primer name	Primer sequence	Primer size (bp)	Tm (°C)	Amplicon expected size (bp)
isotig42940_1F	GAGGCTGATCCCAAGAGATAAT	23	60.3	102
isotig42940_1R	AAATTGTACTGGCCAAAGTGT	22	55.5	
isotig18002_F	AACCAGTGCTAAAGTCCTTGAT	22	58.4	164
isotig18002_R	CTGCTAGCAATGGTGGTCAT	20	58.4	
isotig37981_F	AGCAGCAAGCAGAGCAA	17	52.4	210
isotig37981_R	CAGATGTGGCCAGGATAGATG	21	61.2	
isotig40670_F	GGATTGTGCTCTCTCTCTTCTG	22	61.2	212
isotig40670_R	GTGTTCATCTGCTCGCTCT	19	57.5	
isotig39554_1F	TCTGGCTGTCGAAGGATCTA	21	58.4	127
isotig39554_1R	GGATGTTCTCCATGGTTGGT	21	58.4	
ecu-miR156a	TTGACAGAAGATAGAGAGCAC	22	57.4	–
ecu-miR156b	GCTCTCTATGCTTCTGTCATC	22	59.4	–
ecu-miR2275a	TTGTTTTTCTCCAATATCTC	21	50.2	–
ecu-mir2275b	TTCAGTTTCCTCTAATATCTC	22	53.5	–
ecu-miR396	TCCACAGGCTTTCTTGAACT	21	56.4	–
ecu-miR8175	TCCCCGGCAACGGCGCCA	19	65.3	–
ecu-miR827	TTAGATGACCATCAGCAAACA	22	55.5	–
ecu-miR894	GTTTCACGTCGGGTTCACCA	21	60.5	–
micro_isotig42940	ACAACTAGCAAGATGGCCCGCGC	24	68.2	–
micro_isotig46613_F	ggcggcgTATATTTTGAAACAG	22	60.1	–
micro_isotig46613_s_loop	GTCGTATCCAGTGCAGGGTCCGAGGTATTCGCACTGGATACGACctccct	50	–	–
micro_isotig28724_F	cggcgGAACTCGTAGAGCTT	20	62.5	–
micro_isotig28724_s_loop	GTCGTATCCAGTGCAGGGTCCGAGGTATTCGCACTGGATACGACcgcggc	50	–	–
URP	CCAGTGCAGGGTCCGAGGT	19	63.6	–

Novel miRNA validation was conducted via stem-loop validation assays [48]. RNA was extracted using a NucleoSpin® miRNA kit (Macherey-Nagel, Düren, Germany). The ImProm-II™ reverse transcription system (Promega) was used to reverse transcribe 20 ng of the small fraction of RNA from OTA-S and Tanganyika spikelets using 50 nM stem-loop RT primer, designed as described in [48] (Table 5). Each 20-μl reaction was incubated in a BioRad Thermocycler for 5 min at 25 °C, 60 min at 42 °C, 15 min at 70 °C (to inactivate the reverse transcriptase), and held at 4 °C. The reaction mixture for qRT-PCR consisted of 0.5 μM of a forward primer specific for each miRNA, 0.5 μM of universal reverse stem-loop [48], 1 μl of RT product, and 7.5 μl of 2x Real Mix (Biodynamics, Buenos Aires, Argentina). The cycling conditions were similar to those used for cDNA amplification (95 °C for 5 min, cycle 95 °C for 5 s, 60 °C for 10 s, and 72 °C for 1 s). A melting curve was produced at the end of each reaction to check the specificity of amplification (ramp up from 65 to 95 °C by 0.2 °C per step, waiting 1 s after each step). The qRT-PCR was performed in technical triplicates of two biological samples from OTA-S and Tanganyika spikelets. The 2^-ΔΔCt^ method [49] was used to normalize transcript levels between cDNA samples. Amplification efficiency was calculated with Rotor-Gene 6000 Series Software 1.7 for the samples and the corresponding internal control. Experiments were conducted on technical triplicates of three biological samples. Differences between mean values were evaluated by Student’s t-tests. *P* values< 0.05 were considered significant.

Validation assays of mRNA targets were conducted, using technical triplicates of two biological replicates. To carry out this purpose, specific primers were designed using Primer3 (http://bioinfo.ut.ee/primer3), synthesized by Macrogen (Seoul, Korea), and assayed using cDNA samples from plants with sexual and apomictic genotypes (Table 5).

A flowchart diagram was done to illustrate the criteria followed in the methodology of the manuscript (Additional file 3: Figure S3).

### Differential expression analyses

Differential expression between two conditions was analyzed using the EdgeR package [50], and *P*-values adjusted using the Benjamini and Hochberg method [51], to control the false discovery rate. The corrected P-value of 0.01 and log2 (fold-change) of 2 were set as the threshold for significant differential expression.

## Supplementary information

**Additional file 1:** **Figure S1.** Quality scores across all bases as a function of their positions in the reads for O2P1, O2P2, T3P1, and T3P2. The graphs are the output of the FastQC (0.11.5) program.

**Additional file 2: Figure S2.** Other quality parameters of the small RNA libraries sequenced on the Illumina platform. a) Presence of adaptors against the position in the read; b) N content against the position in the read (%); c) theoretical (blue line) and actual (red line) GC distribution across all sequences (%); d) sequence duplication level. For clarity, only the graphs obtained for the O2P1 library are shown as representatives of the other three libraries. The graphs are the output of FastQC.

**Additional file 3:** **Figure S3.** A flowchart diagram. The criteria followed to analyze is the data is shown.

**Additional file 4:** **Table S1.** Major conserved miRNA families identified in small RNA libraries O2P1, O2P1, T3P1, and T3P2. The last file shows the total number of miRNAs identified from these families and the percentage of miRNAs from these families compared to the total number of miRNAs characterized.

## Data Availability

The datasets generated for this study can be found in NCBI, BioProject PRJNA378998 “*Eragrostis curvula* sRNA raw sequence reads”, including Biosamples SAMN06564601 (biological replicates of reads from Tanganyika) and SAMN06564600 (biological replicates of reads from OTA-S).

## Abbreviations

AGO: Argonaute
EST: Expressed sequence tag
hetsiRNA: heterochromatic siRNA
hp-siRNA: hairpin-derived siRNA
LogFC: Logarithm fold change
miRNA: microRNA
mRNA: messenger RNA
natsiRNA: natural antisense siRNA
O2P1 and O2P2: RNA sampled from OTA, plants 1 and 2, respectively
pre-miRNAs: Stem-loop precursor microRNAs
pri-miRNA: primary microRNA
qRT-PCR: Real-time reverse transcription-PCR
RT-PCR: Reverse transcription polymerase chain reaction
siRNA: small-interfering RNA
SPL: Squamosa-promoter binding-like protein
sRNA: small RNA
T3P1 and T3P2: RNA sampled from Tanganyika, plants 1 and 2, respectively
