# Supplementary figures and images for: Characterization and discovery of miRNA and miRNA targets from apomictic and sexual genotypes of Eragrostis curvula
Source: BMC Genomics. 2019 Nov 12;20:839. doi: 10.1186/s12864-019-6169-0 (PMC6852985; doi:10.1186/s12864-019-6169-0)

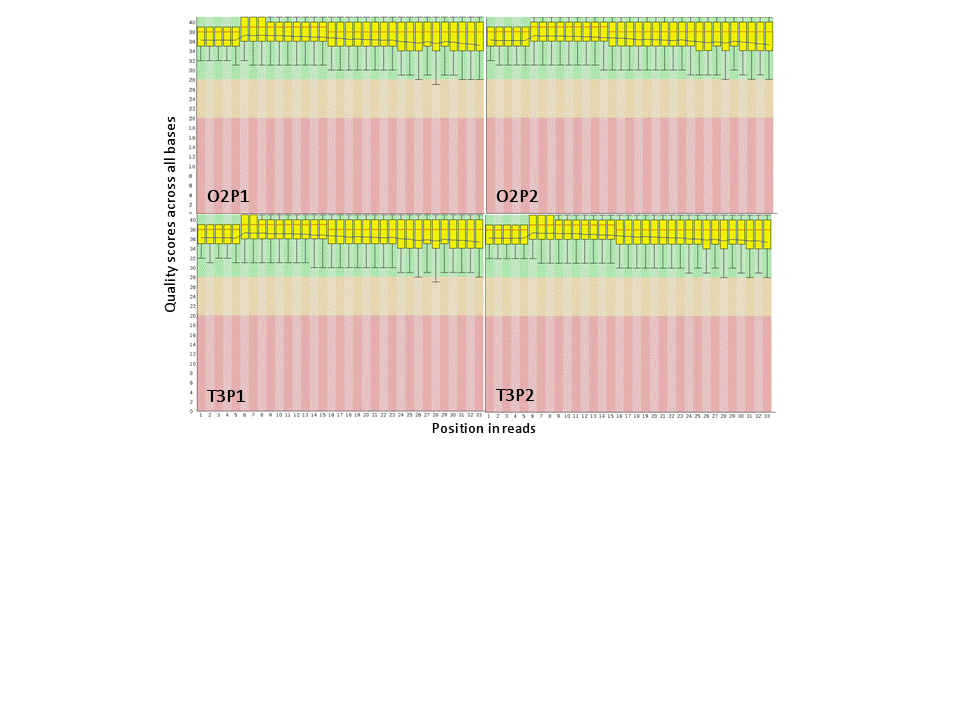

Supplement: Supplementary file 1 — Additional file 1: Figure S1. Quality scores across all bases as a function of their positions in the reads for O2P1, O2P2, T3P1, and T3P2. The graphs are the output of the FastQC (0.11.5) program. [file 12864_2019_6169_MOESM1_ESM.gif]

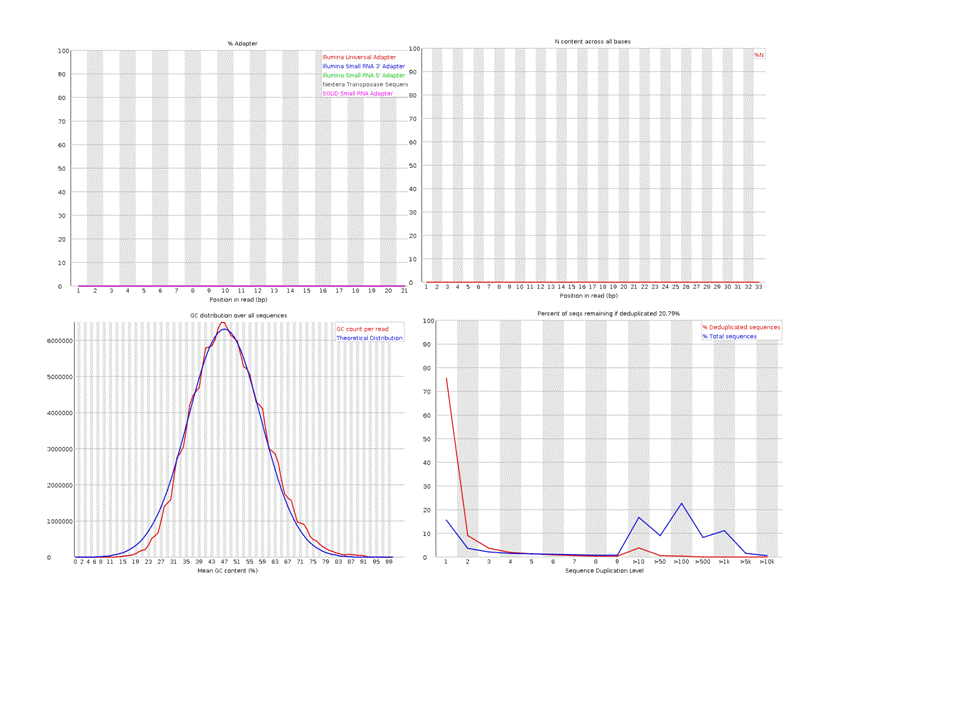

Supplement: Supplementary file 2 — Additional file 2: Figure S2. Other quality parameters of the small RNA libraries sequenced on the Illumina platform. a) Presence of adaptors against the position in the read; b) N content against the position in the read (%); c) theoretical (blue line) and actual (red line) GC distribution across all sequences (%); d) sequence duplication level. For clarity, only the graphs obtained for the O2P1 library are shown as representatives of the other three libraries. The graphs are the output of FastQC. [file 12864_2019_6169_MOESM2_ESM.gif]

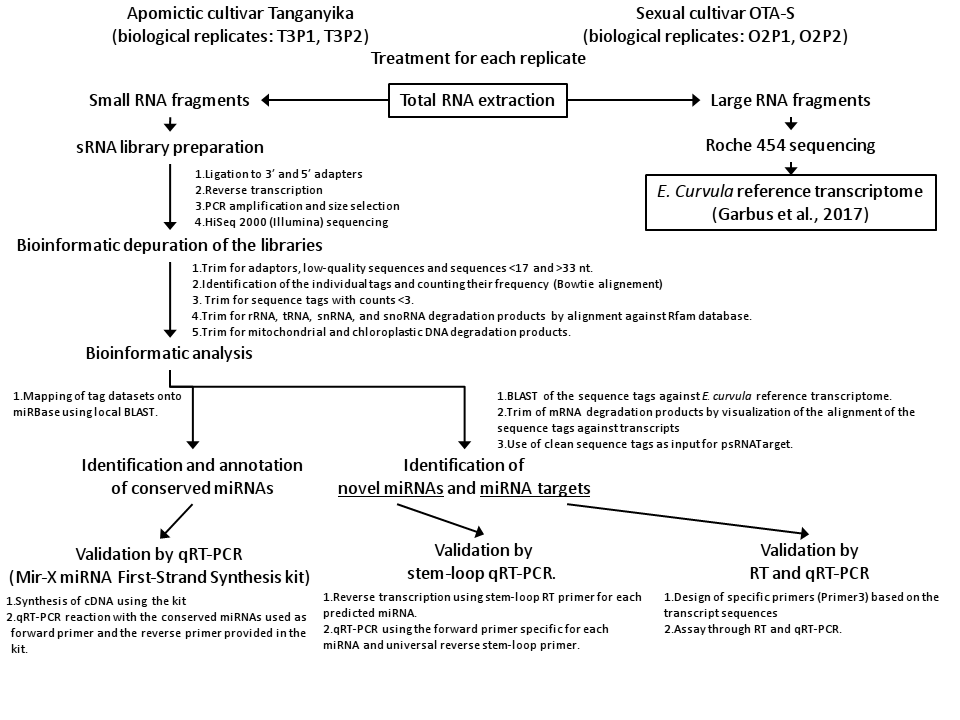

Supplement: Supplementary file 3 — Additional file 3: Figure S3. A flowchart diagram. The criteria followed to analyze is the data is shown. [file 12864_2019_6169_MOESM3_ESM.tif]
