# Supplementary material for: Characterization and discovery of miRNA and miRNA targets from apomictic and sexual genotypes of Eragrostis curvula
Source: BMC Genomics. 2019 Nov 12;20:839. doi: 10.1186/s12864-019-6169-0 (PMC6852985; doi:10.1186/s12864-019-6169-0)
Supplement: Supplementary file 4 — Additional file 4: Table S1. Major conserved miRNA families identified in small RNA libraries O2P1, O2P1, T3P1, and T3P2. The last file shows the total number of miRNAs identified from these families and the percentage of miRNAs from these families compared to the total number of miRNAs characterized. [file 12864_2019_6169_MOESM4_ESM.docx]

**Table S1.** Major conserved miRNAs families identified in the small RNAs libraries O2P1, O2P1, T3P1 and T3P2. The last file totalizes the count of identified miRNAs from these families and shows the percentage of miRNAs from this families as compared to the total miRNAs characterized.

| **miRNA** | **O2P1** | **O2P2** | **T3P1** | **T3P2** |
| --- | --- | --- | --- | --- |
| miR2275 | 20732 | 9827 | 19832 | 18669 |
| miR156 | 11247 | 6944 | 14872 | 18610 |
| MIR894 | 5559 | 5717 | 5847 | 5095 |
| mir396 | 7632 | 2889 | 5876 | 5105 |
| MIR827 | 2350 | 1709 | 3684 | 4663 |
| MIR169 | 5437 | 2465 | 1440 | 1610 |
| miR5072 | 2648 | 1543 | 2261 | 2239 |
| miR-2898 | 1153 | 1351 | 3358 | 2351 |
| miR8175 | 1779 | 950 | 2727 | 2250 |
| mir11341 | 1229 | 795 | 2364 | 2413 |
| miR3711 | 1483 | 1071 | 1577 | 2103 |
| mir166 | 1908 | 1016 | 1297 | 1353 |
| MIR164 | 1574 | 844 | 1555 | 1502 |
| mir-9993 | 997 | 1354 | 1951 | 1051 |
| miR-2478 | 1361 | 1424 | 1656 | 865 |
| MIR319 | 1708 | 1378 | 717 | 813 |
| mir-9 | 2589 | 1438 | 213 | 308 |
| miR5054 | 876 | 792 | 1265 | 1536 |
| MIR444 | 734 | 280 | 1545 | 1707 |
| mir-2796 | 1994 | 1892 | 4 | 3 |
| MIR171 | 991 | 424 | 1072 | 1244 |
| miR-9277 | 782 | 516 | 1224 | 1074 |
| MIR167 | 1448 | 573 | 725 | 735 |
| miR6478 | 333 | 734 | 1611 | 673 |
| miR5139 | 485 | 616 | 1066 | 1042 |
| mir479 | 1347 | 541 | 327 | 742 |
| MIR812 | 481 | 318 | 827 | 1198 |
| miR11338 | 160 | 124 | 1299 | 1237 |
| MIR9774 | 1142 | 618 | 897 | 147 |
| MIR394 | 556 | 499 | 683 | 904 |
| MIR824 | 898 | 695 | 465 | 495 |
| mir-6093 | 753 | 298 | 766 | 627 |
| MIR395 | 341 | 198 | 866 | 935 |
| MIR168 | 494 | 277 | 581 | 826 |
| MIR3454 | 649 | 575 | 403 | 478 |
| mir399 | 407 | 224 | 666 | 660 |
| mir-4335 | 270 | 251 | 595 | 798 |
| MIR4359a | 443 | 417 | 483 | 494 |
| miR11342 | 806 | 323 | 422 | 259 |
| mir-2248 | 675 | 460 | 208 | 410 |
| mir-7 | 173 | 94 | 584 | 787 |
| miR172 | 256 | 162 | 595 | 623 |
| mir-11923 | 0 | 0 | 874 | 730 |
| MIR408 | 337 | 151 | 474 | 526 |
| Mir160 | 333 | 225 | 466 | 415 |
| MIR398 | 342 | 247 | 419 | 410 |
| mir-146 | 18 | 4 | 638 | 714 |
| MIR9767 | 437 | 414 | 241 | 205 |
| MIR8745 | 0 | 0 | 640 | 634 |
| mir-455 | 708 | 562 | 0 | 3 |
| mir-6409 | 235 | 117 | 496 | 404 |
| MIR6300 | 341 | 188 | 265 | 452 |
| mir-8979 | 0 | 0 | 634 | 594 |
| MIR1866 | 141 | 121 | 357 | 514 |
| mir-8525 | 0 | 0 | 432 | 693 |
| mir-12085 | 310 | 234 | 281 | 296 |
| MIR818 | 80 | 39 | 342 | 610 |
| mir-1388 | 254 | 168 | 326 | 276 |
| MIR6024 | 232 | 124 | 275 | 378 |
| mir-3715 | 311 | 264 | 212 | 211 |
| miR5179 | 435 | 209 | 106 | 150 |
| mir-2373 | 195 | 55 | 264 | 364 |
| MIR914 | 130 | 171 | 567 | 5 |
| MIR5485 | 0 | 0 | 353 | 493 |
| MIR8668 | 0 | 0 | 363 | 453 |
| MIR5168 | 390 | 42 | 119 | 260 |
| MIR11169 | 195 | 126 | 306 | 174 |
| MIR3628 | 241 | 138 | 194 | 227 |
| let-7 | 92 | 60 | 263 | 378 |
| mir-1329 | 492 | 300 | 0 | 0 |
| mir-517b | 0 | 0 | 289 | 445 |
| MIR5149 | 406 | 203 | 16 | 7 |
| mir-8877 | 110 | 107 | 365 | 0 |
| MIR904b | 44 | 45 | 357 | 40 |
| **total miRNAs** | **95689** | **58930** | **101340** | **101695** |
| **% of the conserved miRNAs** | **76,7** | **77,5** | **77,1** | **74,6** |
